# Supplementary material for: Tuning the activity and selectivity of electroreduction of CO2 to synthesis gas using bimetallic catalysts
Source: Nat Commun. 2019 Aug 19;10:3724. doi: 10.1038/s41467-019-11352-0 (PMC6700200; doi:10.1038/s41467-019-11352-0)
Supplement: Supplementary file 1 — Supplementary Information [file 41467_2019_11352_MOESM1_ESM.pdf]

Supplementary Information

Tuning the Activity and Selectivity of Electroreduction of CO_2 to Synthesis Gas using Bimetallic Catalysts

Chen *et al.*

Supplementary Figures

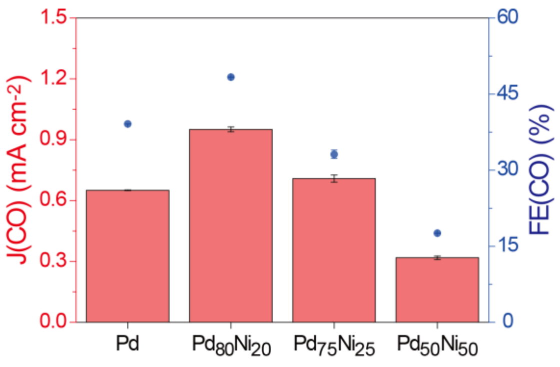

Supplementary Figure 1. The trends of partial current density (J(CO)) and Faradaic efficiency (FE(CO)) of CO at -0.9 V_{RHE} with different bimetallic ratios of Pd and Ni.

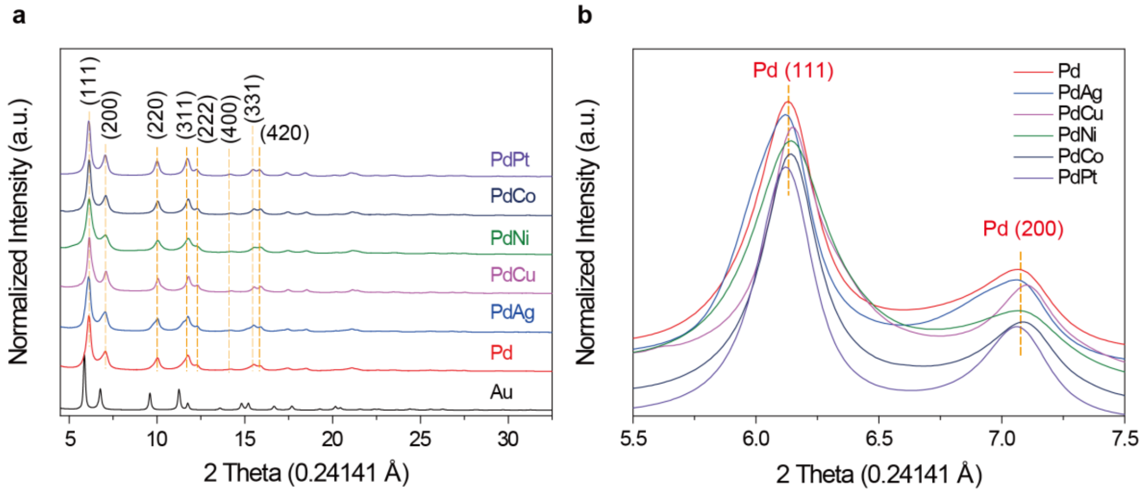

Supplementary Figure 2. Synchrotron powder X-ray diffraction analyses. **a** Powder X-ray diffraction (XRD) patterns for all the samples in this study. **b** Magnified XRD patterns in the 2θ range from 5.5 to 7.5º.

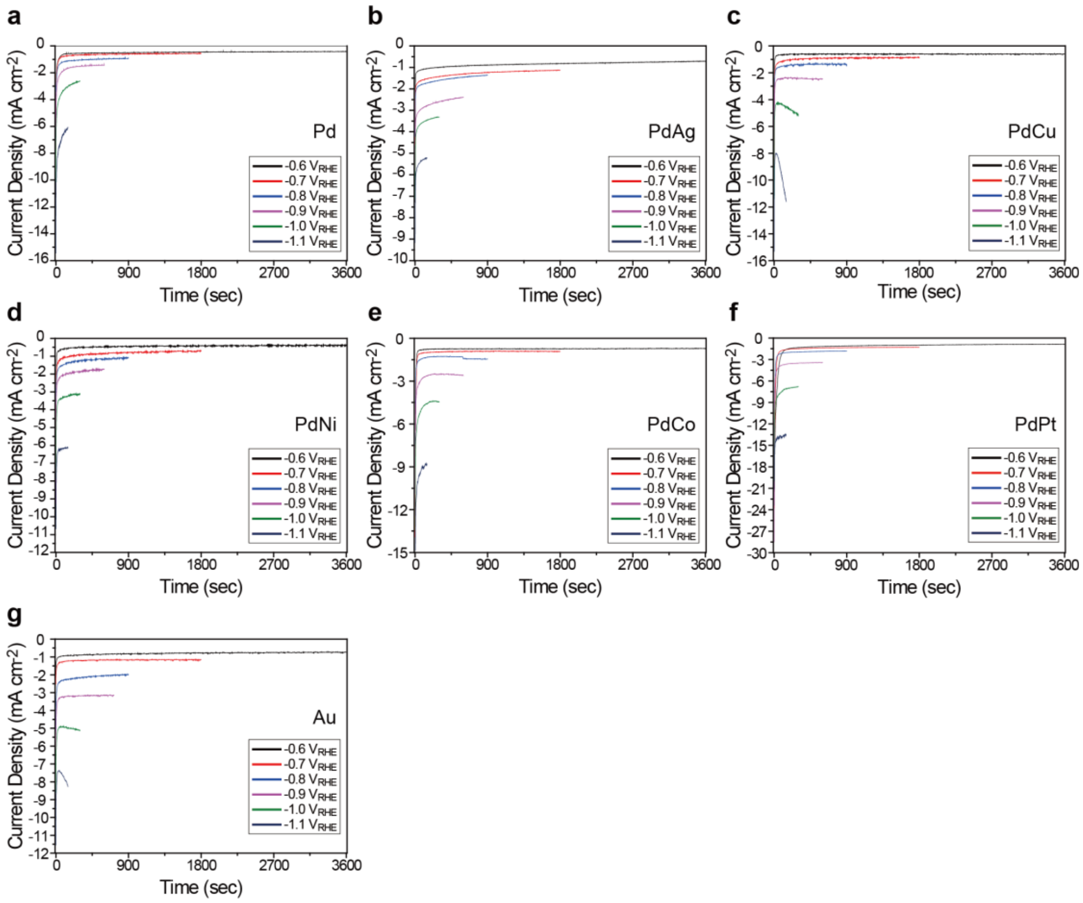

Supplementary Figure 3. Chronoamperometry curves for different catalysts. **a** Pd, **b** PdAg, **c** PdCu, **d** PdNi, **e** PdCo, **f** PdPt, and **g** Au.

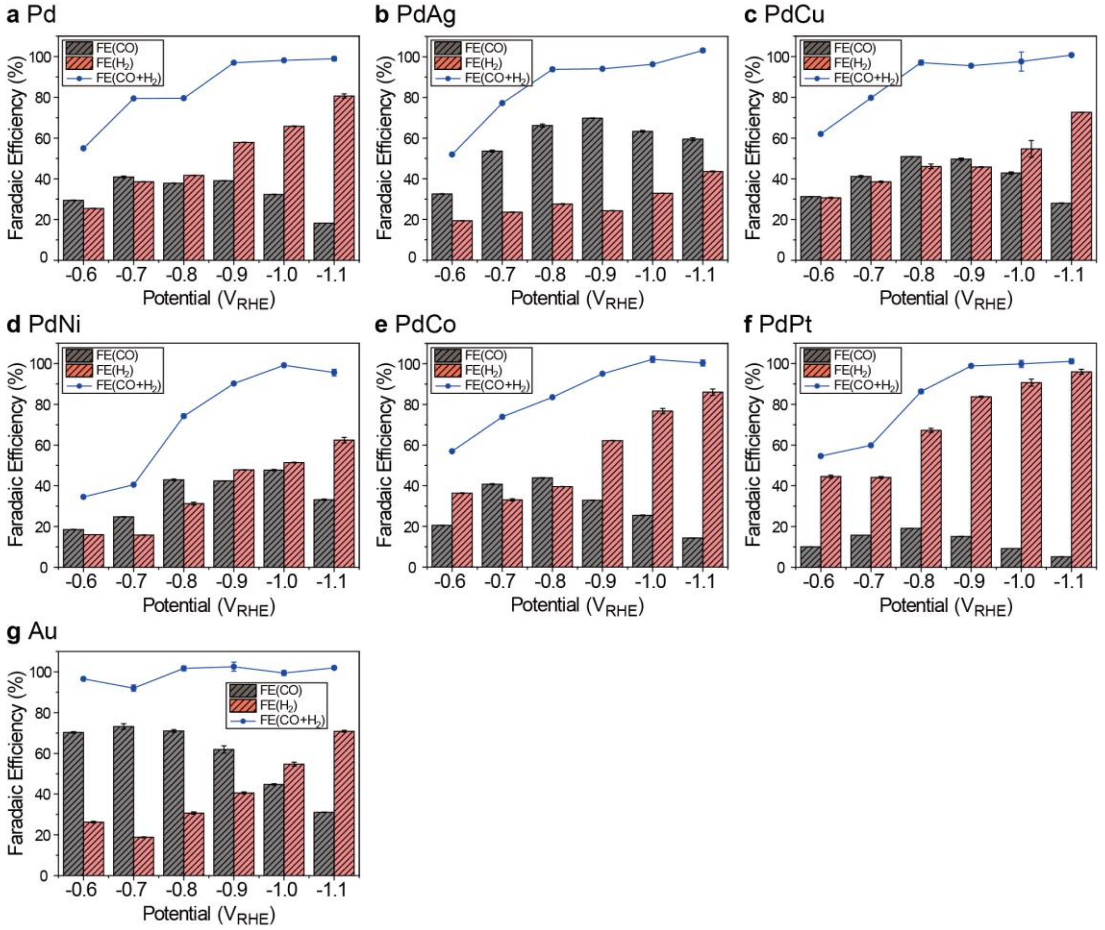

Supplementary Figure 4. Faradaic efficiency of CO (FE(CO)) and H_2 (FE(H_2)) for different catalysts. **a** Pd, **b** PdAg, **c** PdCu, **d** PdNi, **e** PdCo, **f** PdPt, and **g** Au.

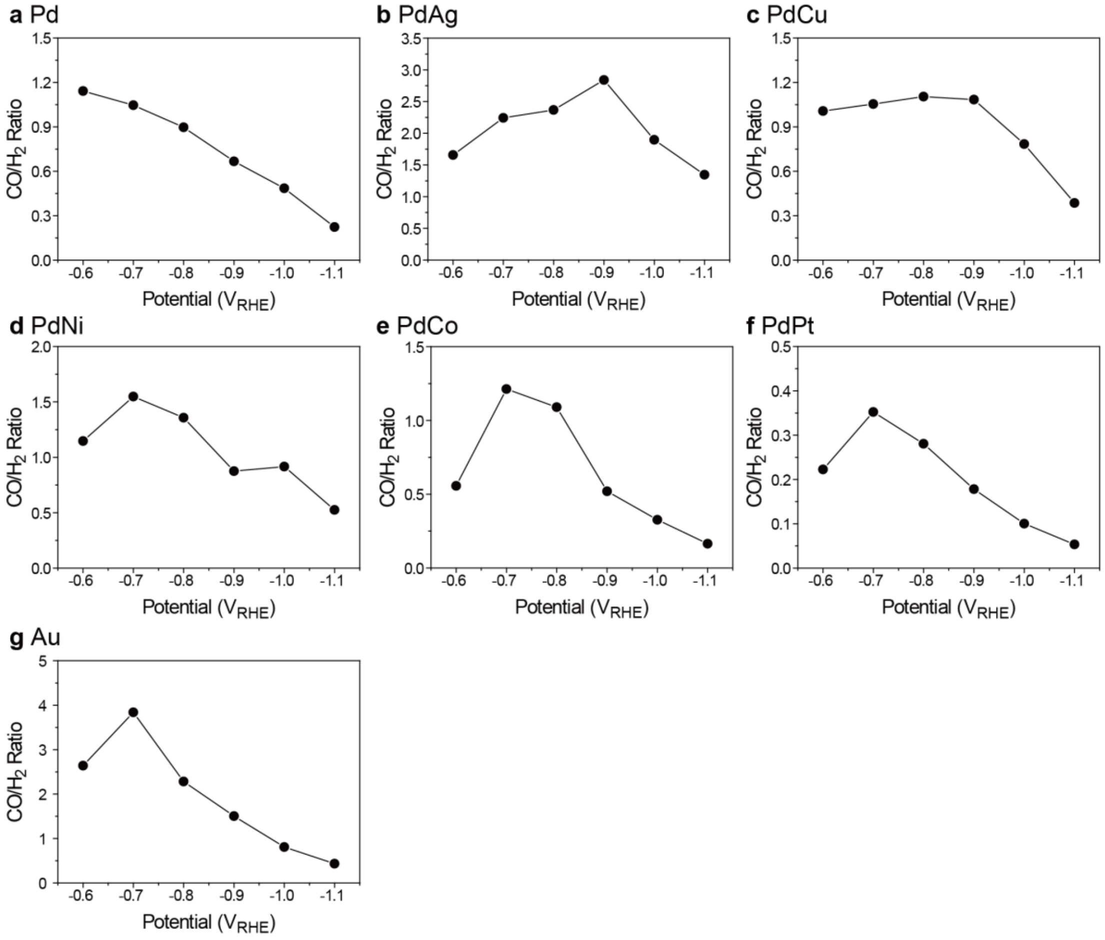

Supplementary Figure 5. Selectivity of CO over H_2 at different potentials for all the samples in this study. **a** Pd, **b** PdAg, **c** PdCu, **d** PdNi, **e** PdCo, **f** PdPt, and **g** Au.

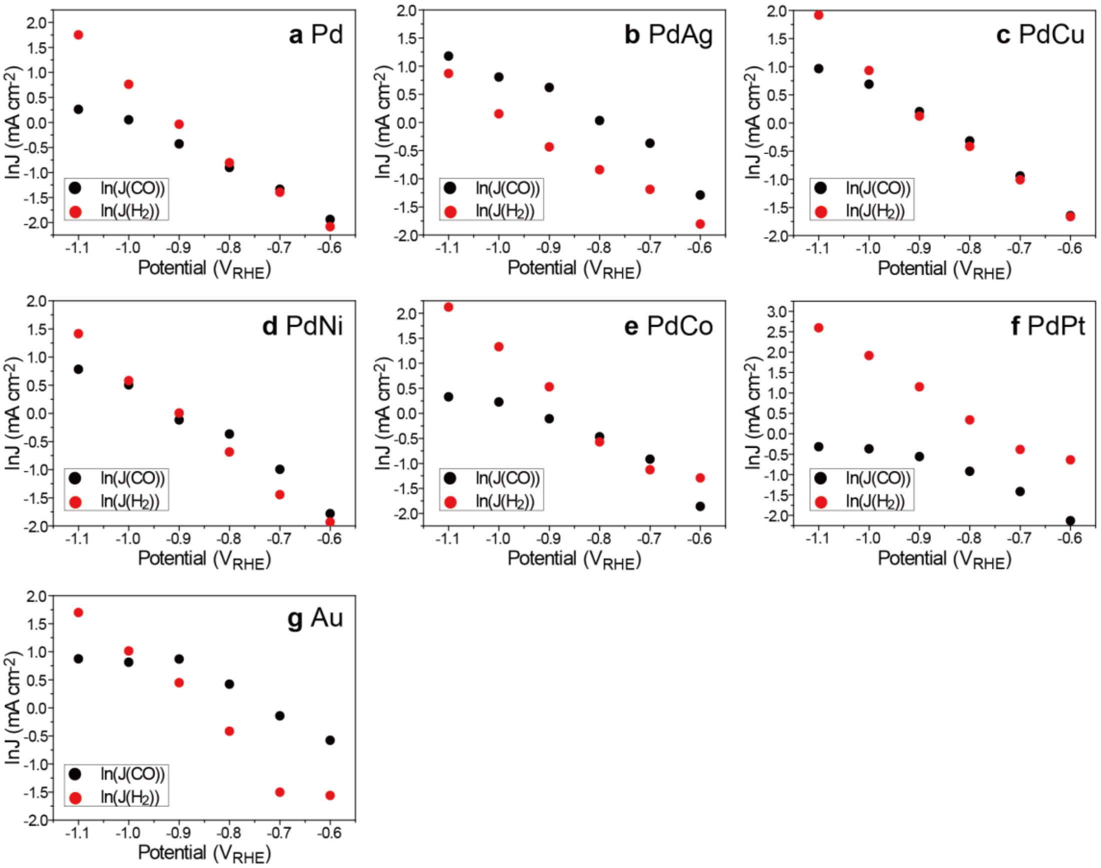

Supplementary Figure 6. Tafel plots of partial current densities of CO and H_2 for all the samples in this study. **a** Pd, **b** PdAg, **c** PdCu, **d** PdNi, **e** PdCo, **f** PdPt, and **g** Au.

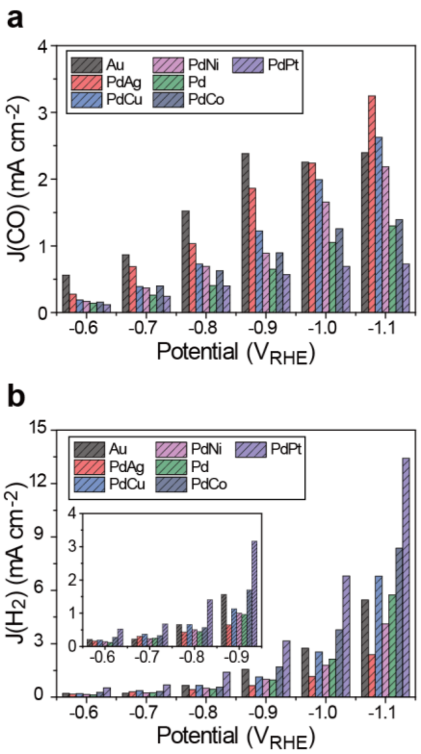

Supplementary Figure 7. Plots of the partial current density versus potential. **a-b** The partial current density of **a** CO (J(CO)) and **b** H_2 (J(H_2)) for all the samples in this study. The inset in **(b)** refers to the magnified range from -0.6 to -0.9 V_{RHE}.

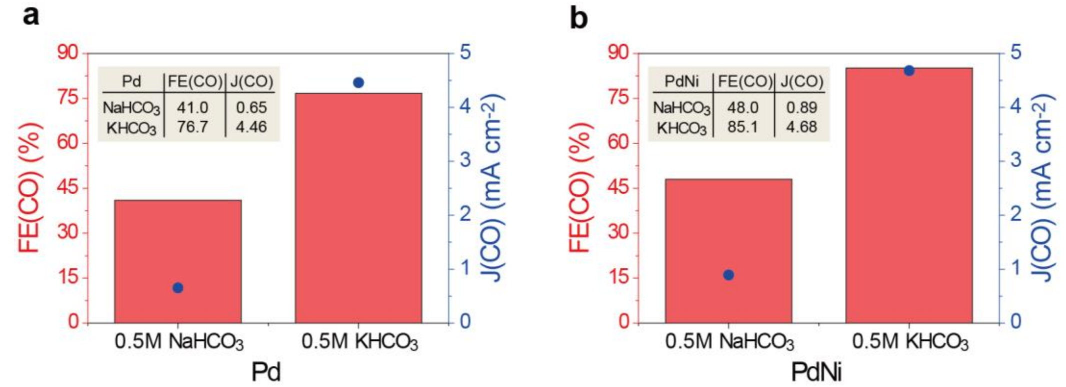

Supplementary Figure 8. Electrocatalytic performance with different electrolytes. **a-b** The partial current density (J(CO)) and Faradaic efficiency (FE(CO)) of CO for **a** Pd and **b** PdNi at -0.9 V_{RHE} in CO_2-saturated 0.5M $NaHCO_3$ and $KHCO_3$ electrolytes.

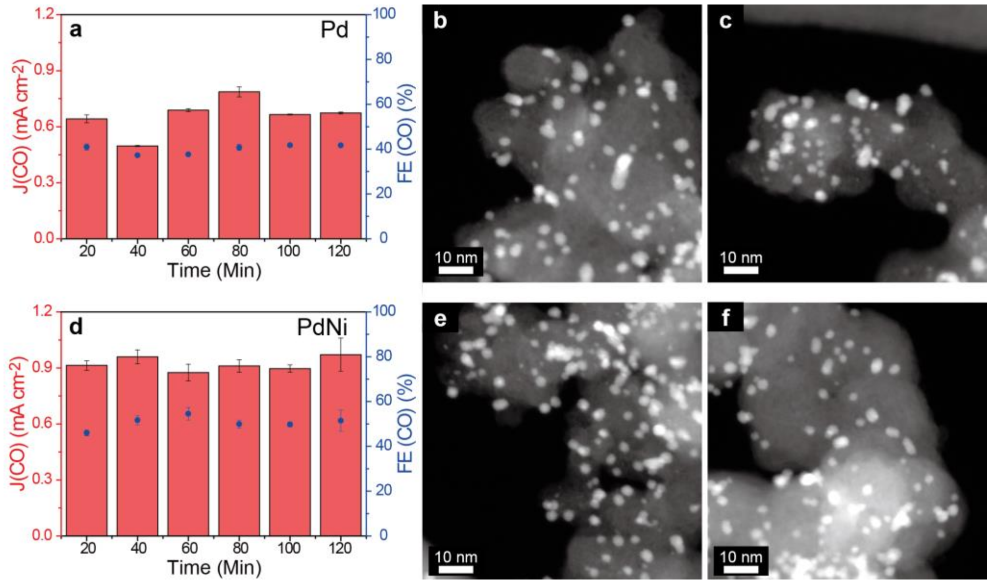

Supplementary Figure 9. Cycling performance of Pd and PdNi. **a** Partial current density (J(CO)) and Faradaic efficiency (FE(CO)) of CO during CO_2RR at -0.9 V_{RHE} with Pd for 2 h. **b-c** STEM images of a Pd catalyst taken **b** before and **c** after CO_2RR. **d-f** The same analyses of a PdNi catalyst.

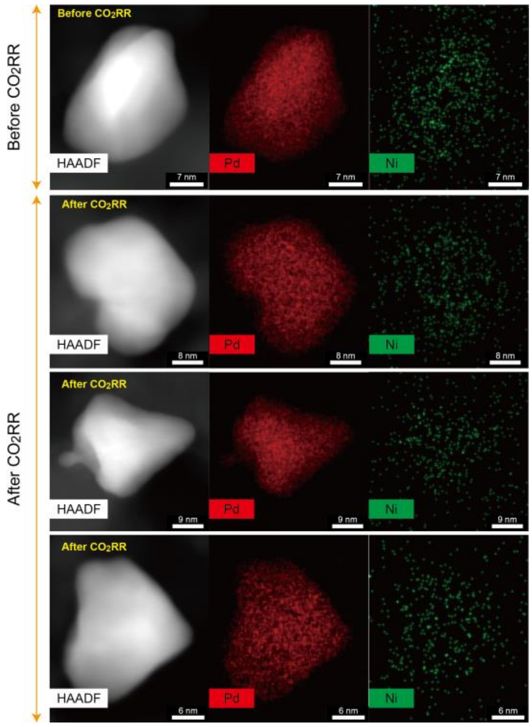

Supplementary Figure 10. STEM-EDX mapping images of Pd (red) and Ni (green) before and after CO_2RR.

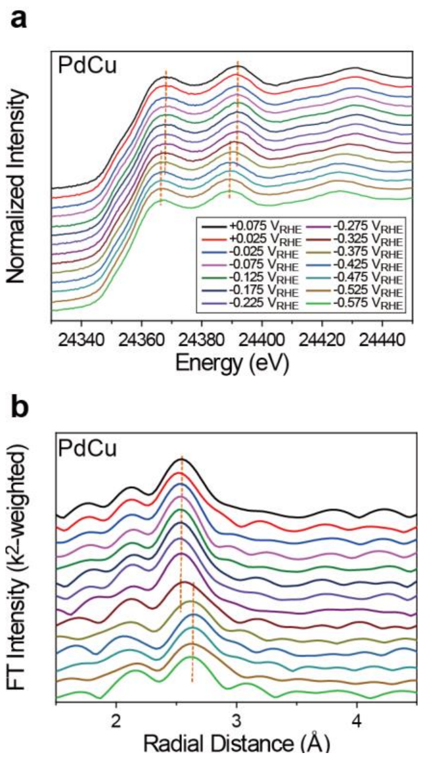

Supplementary Figure 11. *In-situ* XAFS measurement of Pd K-edge for PdCu. **a** XANES spectra and **b** EXAFS spectra. The yellow dotted lines are vertical lines for visualizing the peak shifts during CO_2RR.

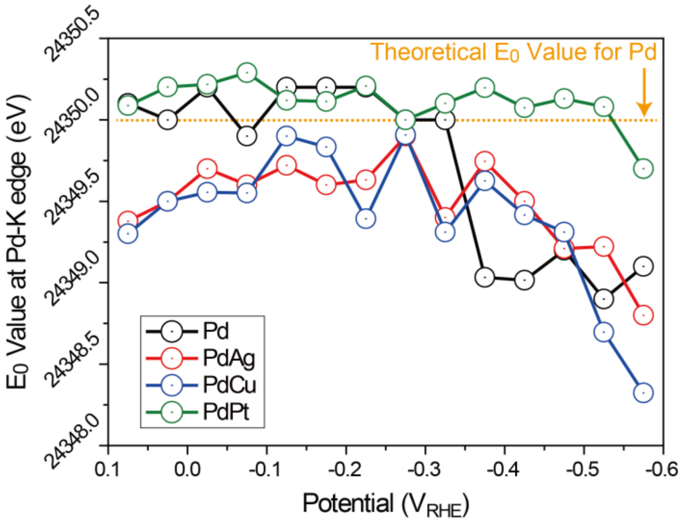

Supplementary Figure 12. Pd K-edge energy (E_0) variation for Pd (black), PdAg (red), PdCu (blue), and PdPt (green) during *in-situ* XAFS analysis. The dotted yellow line indicates the theoretical E_0 value (24350 eV) of Pd.

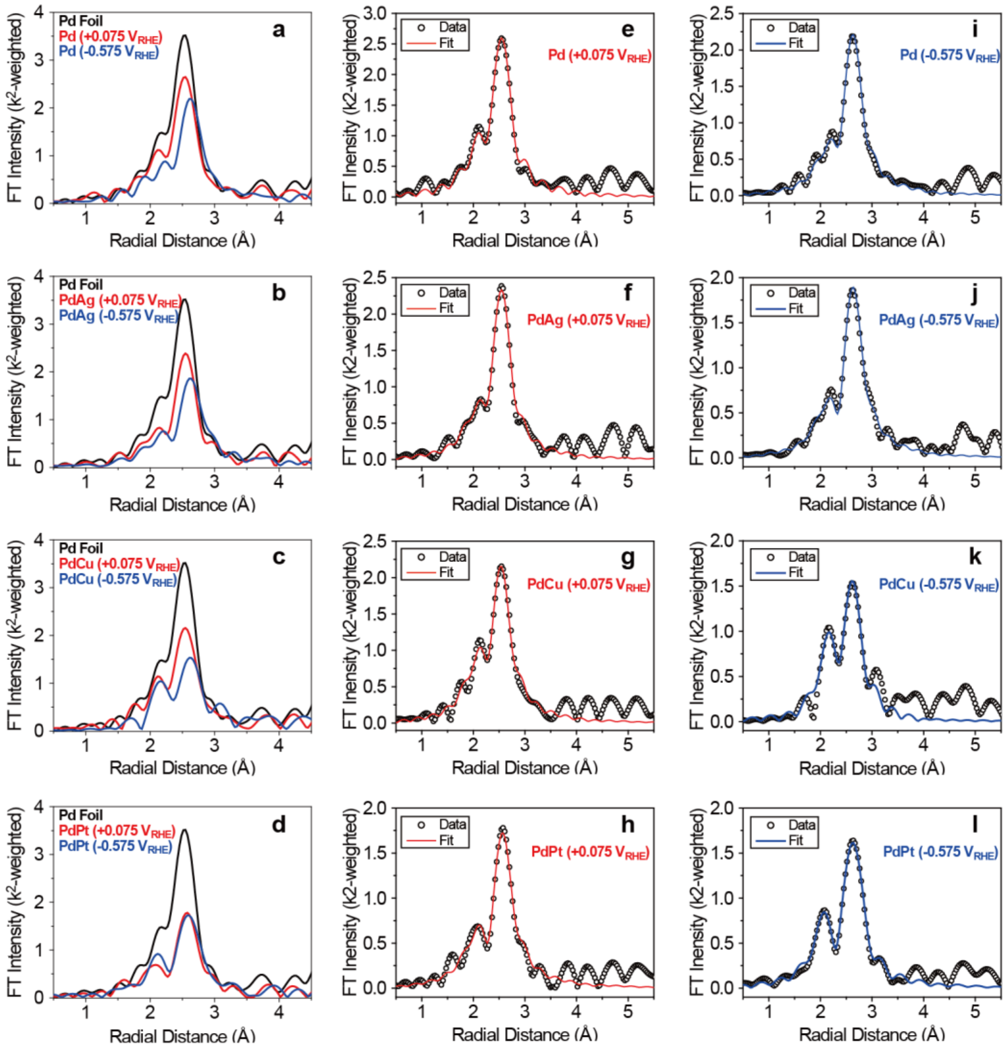

Supplementary Figure 13. EXAFS profiles for Pd K-edge. The profiles for **a** Pd, **b** PdAg, **c** PdCu, and **d** PdPt. Their EXAFS fitting results at **e-h** 0.075 V_{RHE} and **i-l** -0.575 V_{RHE}. The fitting parameters are tabulated in Supplementary Table 2-10.

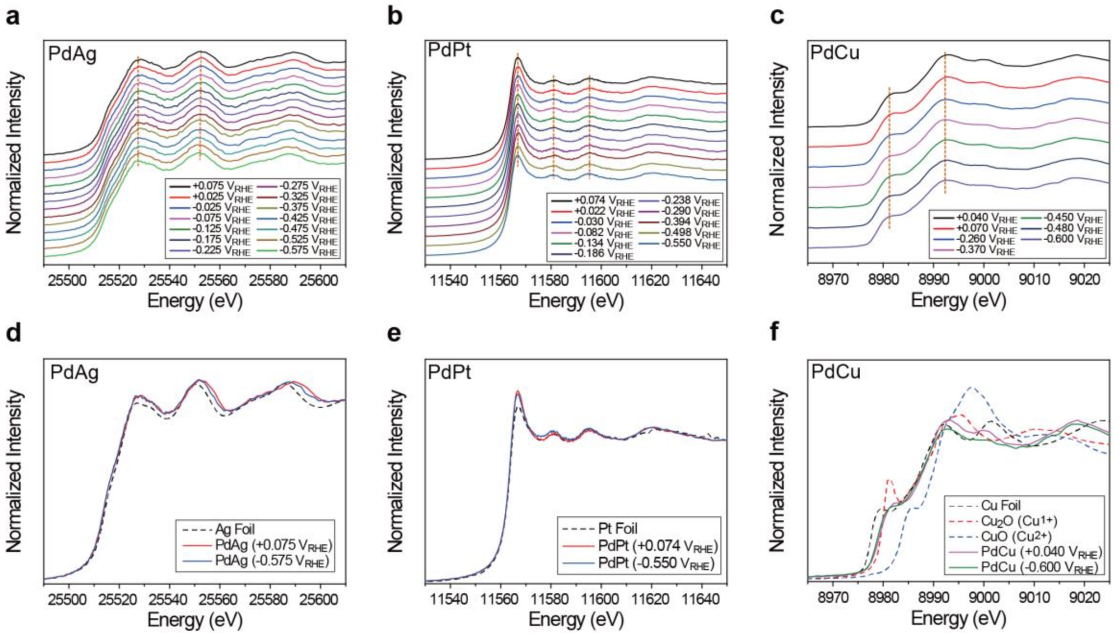

Supplementary Figure 14. *In-situ* XANES data measured at the second metal edges. **a, d** Ag K-edge, **b, e** Pt L_3-edge, and **c, f** Cu K-edge. The yellow dotted lines in **a-c** are vertical lines for visualizing the negligible oxidation state changes during CO_2RR.

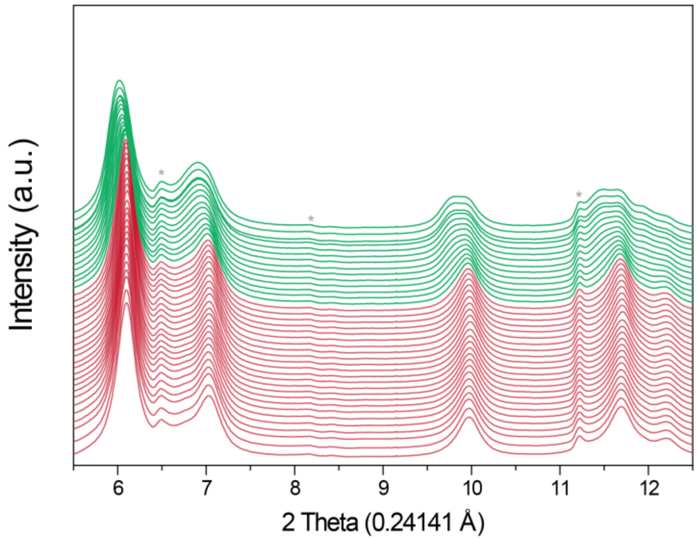

Supplementary Figure 15. *In-situ* X-ray diffraction analyses for PdPt. The analyses were conducted from 0.2 to -0.7 V_{RHE} with a scan rate of 0.05 mV s^{-1}. Interval of each pattern is 24 mV. Red line indicates Pd phase. Green lines indicate their two phase (Pd and PdH) regions. Asterisk (*) marks point out the peaks from the carbon substrate.

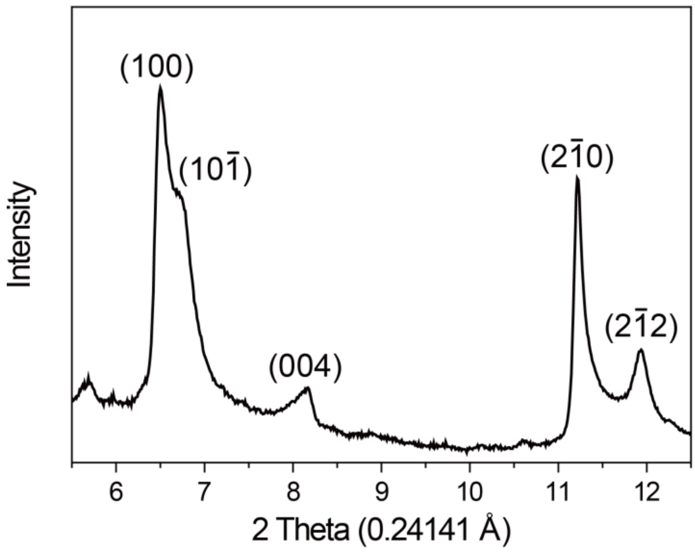

Supplementary Figure 16. X-ray diffraction pattern for Toray carbon paper. The lattice indexing is based on the graphite structure.

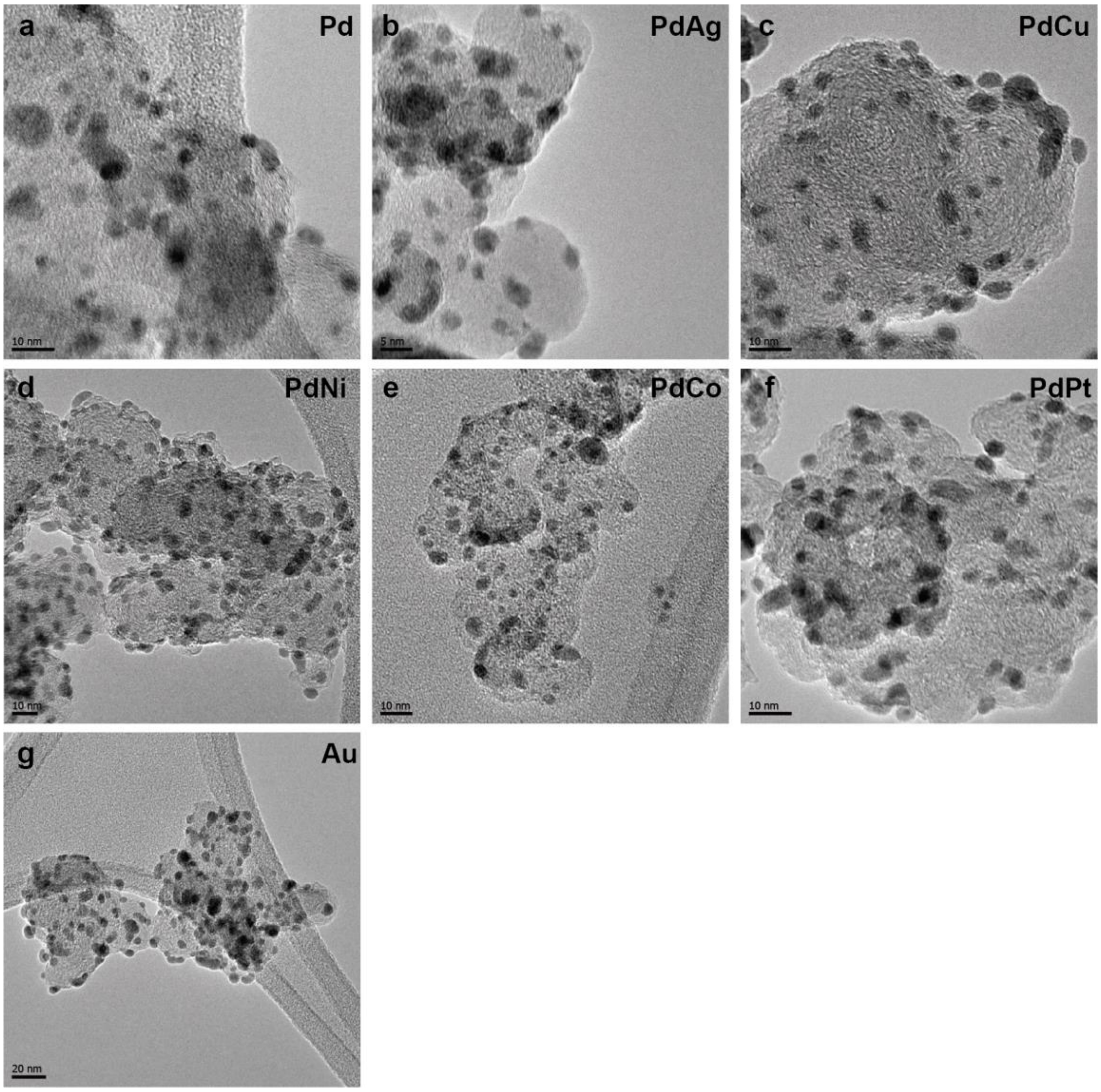

Supplementary Figure 17. TEM images for all the samples in this study. **a** Pd, **b** PdAg, **c** PdCu, **d** PdNi, **e** PdCo, **f** PdPt, and **g** Au.

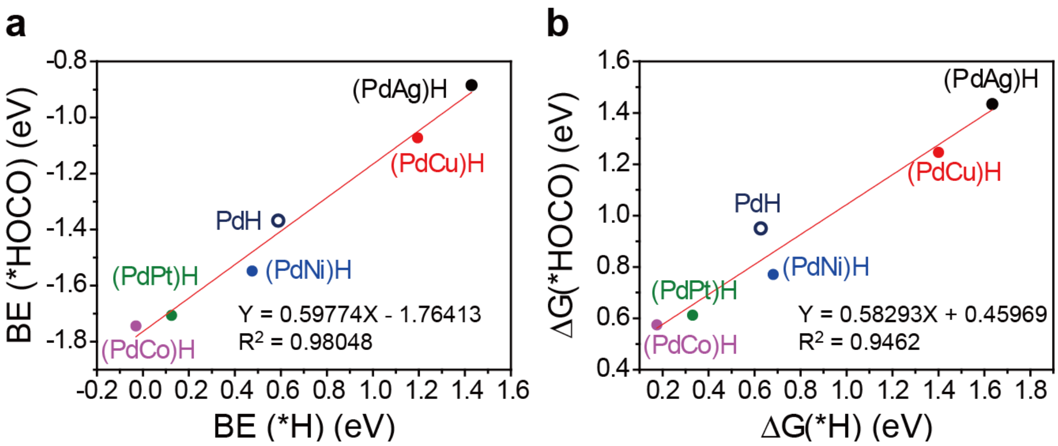

Supplementary Figure 18. Scaling relationships on different catalysts. The correlation of **a** binding energies and **b** free energies (ΔG) between adsorbed *H and *HOCO.

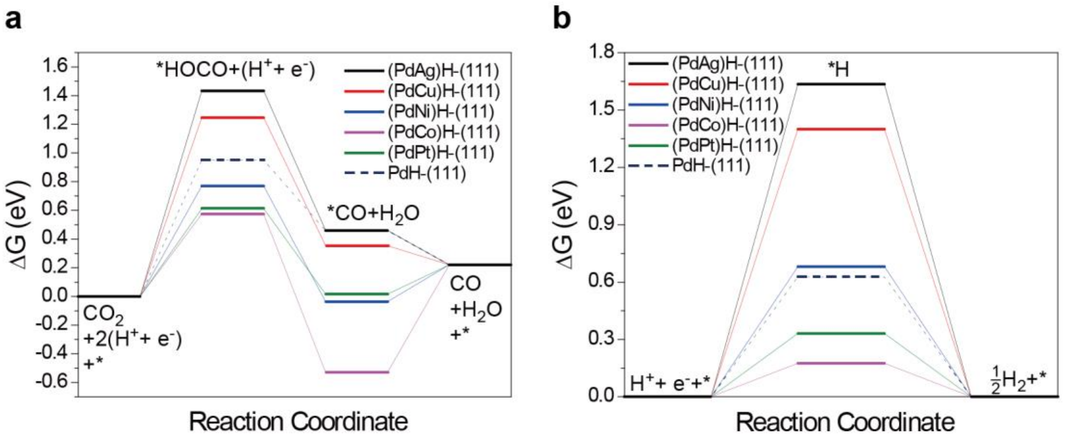

Supplementary Figure 19. DFT calculated free energy diagrams. The free energy (ΔG) diagrams for **a** CO_2RR and **b** HER.

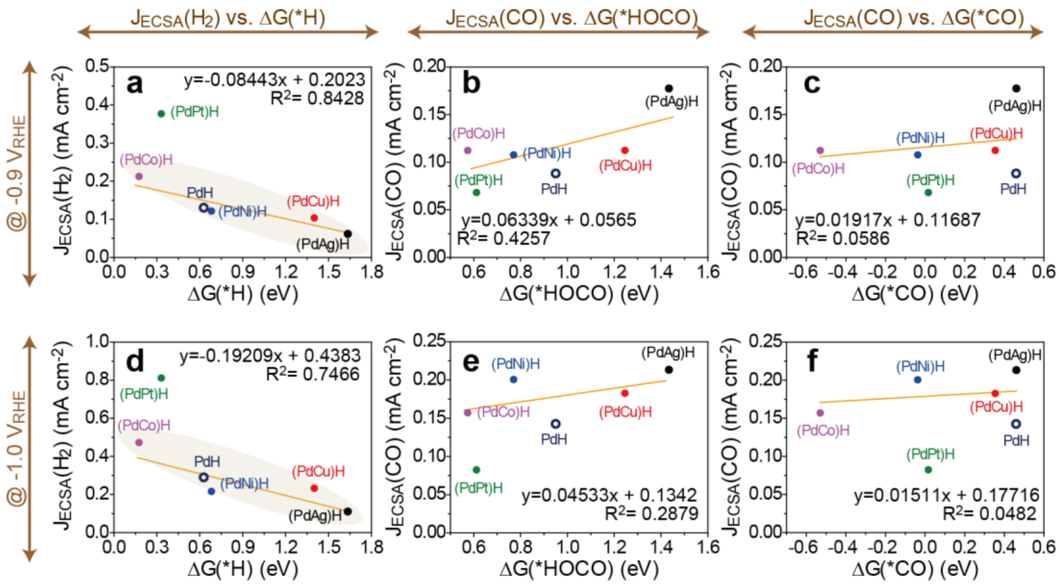

Supplementary Figure 20. Correlations between free energies of each reaction intermediate and the corresponding J_{ECSA}. Correlations at **a-c** -0.9 V_{RHE} and **d-f** -1.0 V_{RHE}.

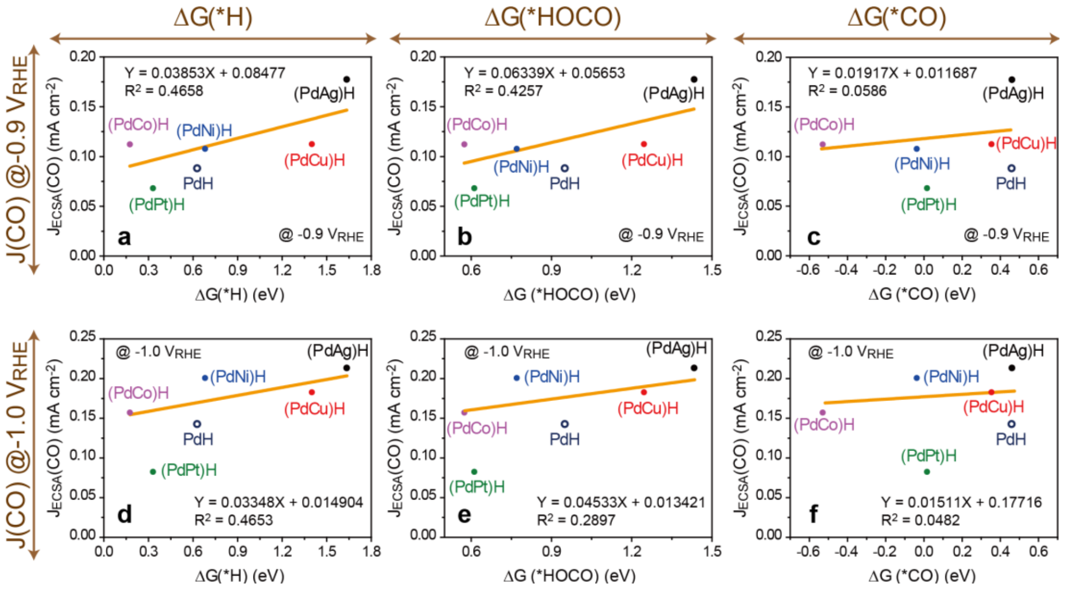

Supplementary Figure 21. Correlations between free energies of each intermediate and J_{ECSA}(CO). **a-c** Correlations between J_{ECSA}(CO) and the free energies of **a** *H, **b** *HOCO, and **c** *CO at -0.9 V_{RHE}. **d-f** The same correlation constructed by using J_{ECSA}(CO) at -1.0 V_{RHE}.

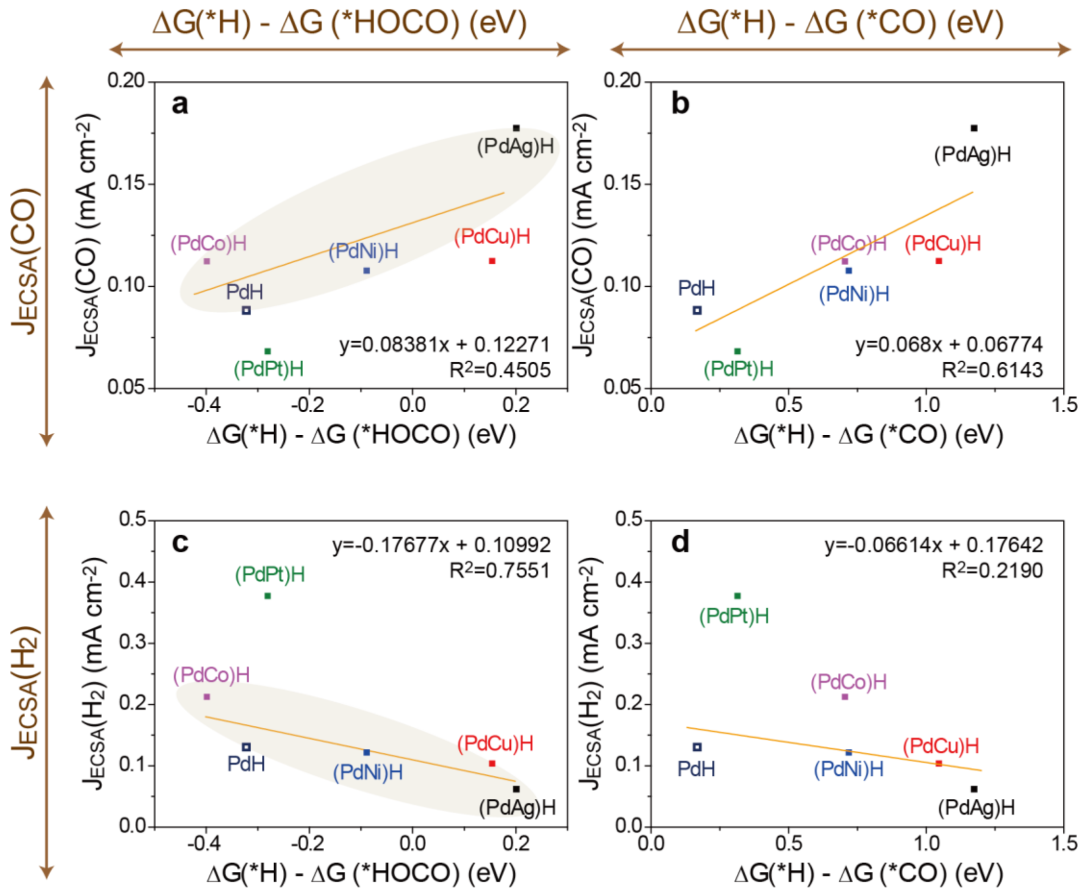

Supplementary Figure 22. Correlations between J_{ECSA} at -0.9 V_{RHE} and free energy difference of reaction intermediates. **a-b** Correlation between $J_{ECSA}(CO)$ and the free energy difference of **a** *H and *HOCO and **b** *H and *CO. **c-d** The same correlation constructed by using $J_{ECSA}(H_2)$.

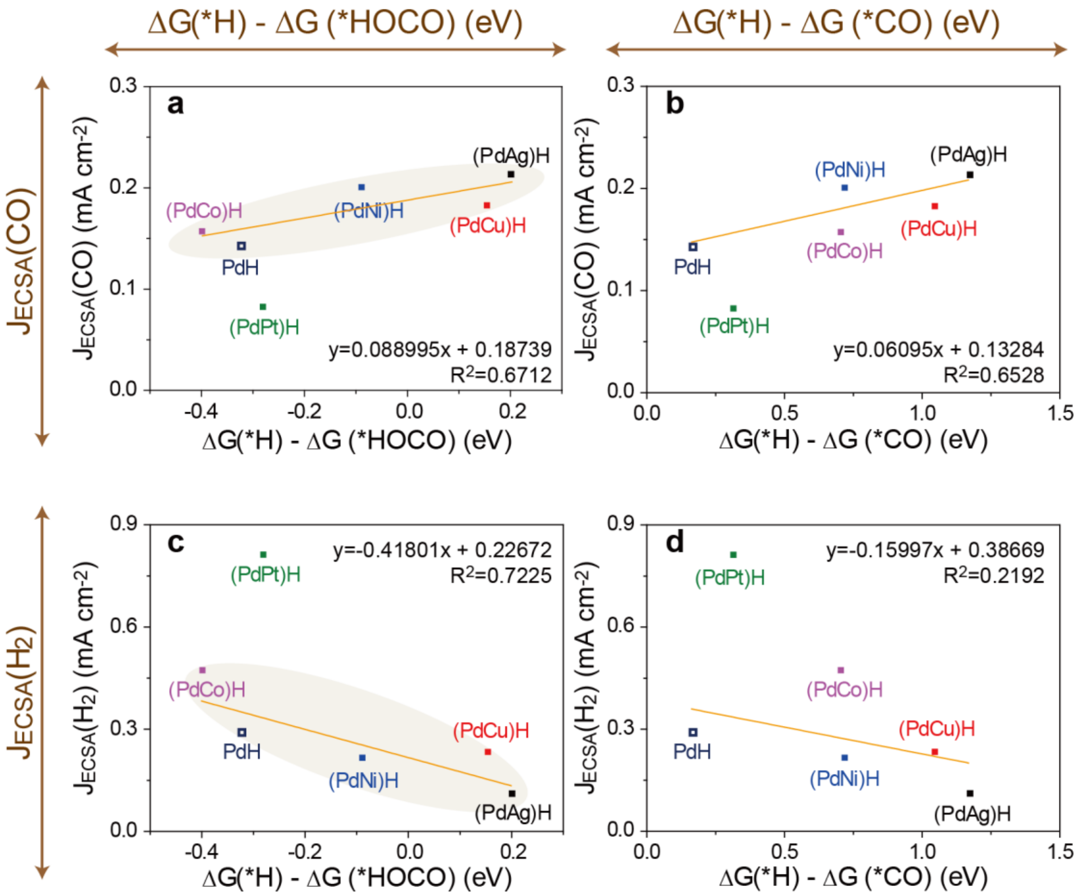

Supplementary Figure 23. Correlations between J_{ECSA} at -1.0 V_{RHE} and free energy difference of reaction intermediates. **a-b** Correlation between J_{ECSA}(CO) and the free energy difference of **a** *H and *HOCO and **b** *H and *CO. **c-d** The same correlation constructed by using $J_{ECSA}(H_2)$

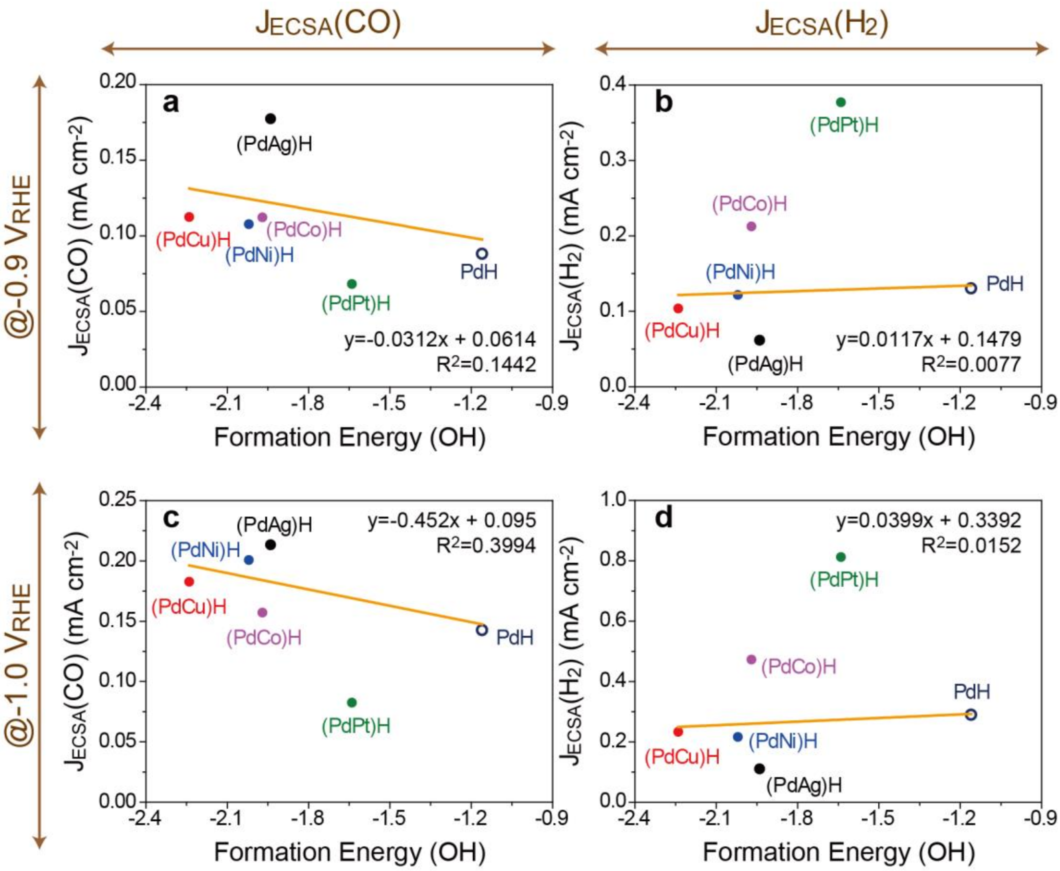

Supplementary Figure 24. Correlations between J_{ECSA} and OH formation energies. **a-b** Correlation using **a** $J_{ECSA}(CO)$ and **b** $J_{ECSA}(H_2)$ at -0.9 V_{RHE}. **c-d** The same correlation constructed by using **c** $J_{ECSA}(CO)$ and **d** $J_{ECSA}(H_2)$ at -1.0 V_{RHE}.

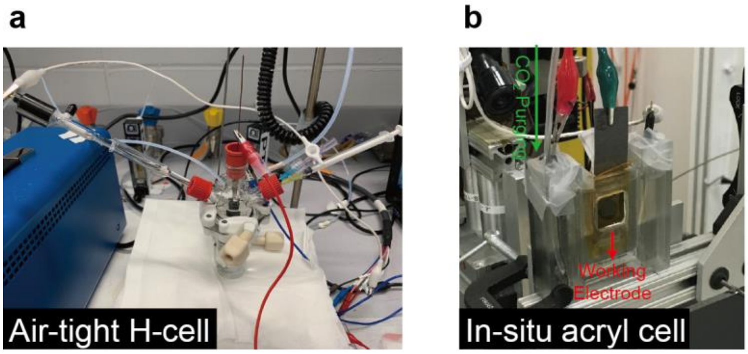

Supplementary Figure 25. The digital photo images for the cell used in this study. **a** Electrochemical cell for electrocatalytic performance evalution. **b** Electrochemical cell used in synchrotron experiments.

Supplementary Tables

Supplementary Table 1. The elemental analysis by ICP-OES for the bimetallic PdM samples in this study.

Entry	Pd (ppm)	M (ppm)	mol ratio of Pd/M[a)]	x in $Pd_{100-x}M_x$
$Pd_{80}Ag_{20}$	0.428	0.105	4.13	19.5
$Pd_{80}Cu_{20}$	1.308	0.201	3.89	20.5
$Pd_{80}Ni_{20}$	1.460	0.205	3.93	20.3
$Pd_{75}Ni_{25}$	1.146	0.254	2.49	28.7
$Pd_{50}Ni_{50}$	1.113	0.534	1.15	46.5
$Pd_{80}Co_{20}$	0.776	0.121	3.55	22.0
$Pd_{80}Pt_{20}$	0.957	0.467	3.76	21.0

[a)] Pd (106.42 g mol^{-1}), Ag (107.868 g mol^{-1}), Cu (63.546 g mol^{-1}), Ni (58.693 g mol^{-1}), Co (58.933 g mol^{-1}), Pt (195.084 g mol^{-1}).

Supplementary Table 2. EXAFS fitting parameters at Pd K-edge for Pd at the potential of 0.075 V_{RHE}.[a)]

Path	Model C.N.[b)]	Fitted C.N.[b)]	R_{eff} (Å)[c)]	R_{fit} (Å)[d)]	σ^2 (Å^2)[e)]
Pd-Pd	12	11.7(4)	2.7505	2.74(1)	0.0070(4)
Independent Points			13.1484375		
Number of Variables			4		
k Range for Fourier Transformation			3.000 - 12.966		
R Range for EXAFS Fitting			1.28 - 3.4		
χ^2			1054.5306755		
Reduced χ^2			115.2689381		
R-factor			0.0069043		

a) Amplitude reduction factor (S_o^2) attained from reference Pd foil was used in EXAFS fitting. Fitting was done in *R*-space.

b) Coordination number (C.N.) for the corresponding scattering path(s).

c) Bond length from the structural model.

d) Bond length from the fitted result.

e) Debye-Waller factor.

Supplementary Table 3. EXAFS fitting parameters at Pd K-edge for Pd at the potential of -0.575 V_{RHE}.[a)]

Path	Model C.N.[b)]	Fitted C.N.[b)]	R_{eff} (Å)[c)]	R_{fit} (Å)[d)]	σ^2 (Å^2)[e)]
Pd-Pd	12	12.0(3)	2.8284	2.826(2)	0.0074(4)
Independent Points			12.6875000		
Number of Variables			4		
k Range for Fourier Transformation			3.000 - 14.277		
R Range for EXAFS Fitting			1.72 - 3.55		
χ^2			533.5934426		
Reduced χ^2			61.4208279		
R-factor			0.0099311		

[a)] Amplitude reduction factor (S_o^2) attained from reference Pd foil was used in EXAFS fitting. Fitting was done in *R*-space.

[b)] Coordination number (C.N.) for the corresponding scattering path(s).

[c)] Bond length from the structural model.

[d)] Bond length from the fitted result.

[e)] Debye-Waller factor.

Supplementary Table 4. EXAFS fitting parameters at Pd K-edge for PdAg at the potential of 0.075 V_{RHE}.[a)]

Path	Model C.N.[b)]	Fitted C.N.[b)]	R_{eff} (Å)[c)]	R_{fit} (Å)[d)]	σ^2 ($Å^2$)[e)]
Pd-M[f)]	12	11.2(6)	2.7505	2.7500(5)	0.0073(4)
Independent Points			12.0312500		
Number of Variables			4		
k Range for Fourier Transformation			3.000 - 14.258		
R Range for EXAFS Fitting			1.656 - 3.356		
χ^2			75.6744430		
Reduced χ^2			9.4224987		
R-factor			0.0085764		

a) Amplitude reduction factor (S_o^2) attained from reference Pd foil was used in EXAFS fitting. Fitting was done in *R*-space.

b) Coordination number (C.N.) for the corresponding scattering path(s).

c) Bond length from the structural model.

d) Bond length from the fitted result.

e) Debye-Waller factor.

f) M=Pd and/or Ag.

Supplementary Table 5. EXAFS fitting parameters at Pd K-edge for PdAg at the potential of -0.575 V_{RHE}.[a)]

Path	Model C.N.[b)]	Fitted C.N.[b)]	R_{eff} (Å)[c)]	R_{fit} (Å)[d)]	σ^2 ($Å^2$)[e)]
Pd-M[f)]	12	11.6(6)	2.8284	2.822(7)	0.0083(4)
Independent Points			12.9062500		
Number of Variables			4		
k Range for Fourier Transformation			3.000 - 14.258		
R Range for EXAFS Fitting			1.68 - 3.500		
χ^2			68.2927019		
Reduced χ^2			7.6679525		
R-factor			0.0106361		

[a)] Amplitude reduction factor (S_o^2) attained from reference Pd foil was used in EXAFS fitting. Fitting was done in *R*-space.

[b)] Coordination number (C.N.) for the corresponding scattering path(s).

[c)] Bond length from the structural model.

[d)] Bond length from the fitted result.

[e)] Debye-Waller factor.

[f)] M=Pd and/or Ag.

Supplementary Table 6. EXAFS fitting parameters at Pd K-edge for PdCu at the potential of 0.075 V_{RHE}.[a)]

Path	Model C.N.[b)]	Fitted C.N.[b)]	R_{eff} (Å)[c)]	R_{fit} (Å)[d)]	σ^2 ($Å^2$)[e)]
Pd-Pd	9.6	9.0(6)	2.7505	2.73(2)	0.009(1)
Pd-Cu	2.4	1.9(1)	2.7505	2.6(1)	0.015(5)
Independent Points			13.7812500		
Number of Variables			6		
k Range for Fourier Transformation			3.000 - 14.267		
R Range for EXAFS Fitting			1.55 - 3.52		
χ^2			44.5418082		
Reduced χ^2			5.72424844		
R-factor			0.0175227		

a) Amplitude reduction factor (S_o^2) attained from reference Pd foil was used in EXAFS fitting. Fitting was done in *R*-space.

b) Coordination number (C.N.) for the corresponding scattering path(s).

c) Bond length from the structural model.

d) Bond length from the fitted result.

e) Debye-Waller factor.

Supplementary Table 7. EXAFS fitting parameters at Pd K-edge for PdCu at the potential of -0.575 V_{RHE}.[a)]

Path	Model C.N.[b)]	Fitted C.N.[b)]	R_{eff} (Å)[c)]	R_{fit} (Å)[d)]	σ^2 ($Å^2$)[e)]
Pd-Pd	9.6	10.1(3)	2.8284	2.78(5)	0.0075
Pd-Cu	2.4	2.53(9)	2.8284	2.7(1)	0.0083
Independent Points			7.2773438		
Number of Variables			4		
k Range for Fourier Transformation			2.349-12.726		
R Range for EXAFS Fitting			1.85-3		
χ^2			164.9189179		
Reduced χ^2			50.3209094		
R-factor			0.0135135		

a) Amplitude reduction factor (S_o^2) attained from reference Pd foil was used in EXAFS fitting. Fitting was done in *R*-space.

b) Coordination number (C.N.) for the corresponding scattering path(s).

c) Bond length from the structural model.

d) Bond length from the fitted result.

e) Debye-Waller factor. In this fitting. σ^2 was fixed due to the limited independent points.

Supplementary Table 8. EXAFS fitting parameters at Pd K-edge for PdPt at the potential of 0.075 V_{RHE}.[a)]

Path	Model C.N.[b)]	Fitted C.N.[b)]	R_{eff} (Å)[c)]	R_{fit} (Å)[d)]	σ^2 (Å^2)[e)]
Pd-Pd	9.6	9.9(5)	2.7505	2.751(10)	0.0085
Pd-Pt	2.4	1.5(1.0)	2.7505	2.71(4)	0.0085
Independent Points			9.1523438		
Number of Variables			5		
k Range for Fourier Transformation			3.000 - 13.731		
R Range for EXAFS Fitting			1.75 - 3.15		
χ^2			48.1183630		
Reduced χ^2			11.6617166		
R-factor			0.0076392		

a) Amplitude reduction factor (S_o^2) attained from reference Pd foil was used in EXAFS fitting. Fitting was done in *R*-space.

b) Coordination number (C.N.) for the corresponding scattering path(s).

c) Bond length from the structural model.

d) Bond length from the fitted result.

e) Debye-Waller factor. In this fitting. σ^2 was fixed due to the limited independent points.

Supplementary Table 9. EXAFS fitting parameters at Pd K-edge for PdPt at the potential of -0.575 V_{RHE}.[a)]

Path	Model C.N.[b)]	Fitted C.N.[b)]	R_{eff} (Å)[c)]	R_{fit} (Å)[d)]	σ^2 ($Å^2$)[e)]
Pd-Pd	9.6	9.6(4)	2.8284	2.76(7)	0.00764
Pd-Pt	2.4	2.5(6)	2.8284	2.74(9)	0.00255
Independent Points			10.2324219		
Number of Variables			5		
k Range for Fourier Transformation			3.000 - 11.5		
R Range for EXAFS Fitting			1.45-3.4		
χ^2			54.9219835		
Reduced χ^2			10.4964746		
R-factor			0.0043668		

a) Amplitude reduction factor (S_o^2) attained from reference Pd foil was used in EXAFS fitting. Fitting was done in *R*-space.

b) Coordination number (C.N.) for the corresponding scattering path(s).

c) Bond length from the structural model.

d) Bond length from the fitted result.

e) Debye-Waller factor. In this fitting. σ^2 was fixed due to the limited independent points.

Supplementary Table 10. EXAFS fitting parameters at Pd K-edge for Pd foil.[a)]

Path	Model C.N.[b)]	Fitted C.N.[b)]	R_{eff} (Å)[c)]	R_{fit} (Å)[d)]	σ^2 ($Å^2$)[e)]
Pd-Pd	12	12.0(5)	2.7505	2.74(1)	0.0058(2)
Independent Points			14.65625		
Number of Variables			4		
k Range for Fourier Transformation			3.000 - 14.258		
R Range for EXAFS Fitting			1.33-3.42		
χ^2			6628.3447371		
Reduced χ^2			622.0147554		
R-factor			0.0043804		

[a)] Amplitude reduction factor (S_o^2) was attained from this EXAFS fitting. Fitting was done in *R*-space.

[b)] Coordination number (C.N.) for the corresponding scattering path(s).

[c)] Bond length from the structural model.

[d)] Bond length from the fitted result.

[e)] Debye-Waller factor.

Supplementary Table 11. DFT calculated lattice parameters (A) and formation energy (ΔE) and unit cell used in calculations.

**Entry**	**A (Å)**	**ΔE (eV)**	**Unit Cell (Pd: gray, M: blue, H: green)**
**(PdAg)H**	4.190	3.58	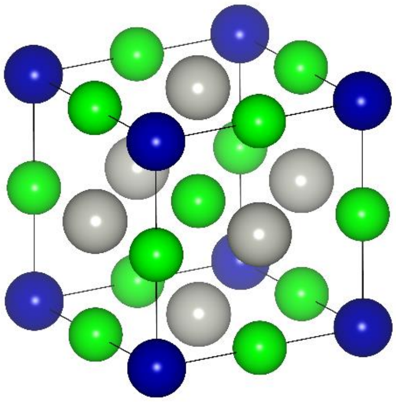
**(PdCu)H**	4.090	1.72	
**(PdNi)H**	4.063	-0.93	
**PdH**	4.145	-0.49	
**(PdCo)H**	4.066	-1.85	
**(PdPt)H**	4.159	-2.78	

Supplementary Table 12. DFT calculated binding energies (BEs, in eV) of *H, *HOCO and *CO on Pd and M sites of PdH and PdMH

Entry	BE(*H)	BE(*HOCO)	BE(*CO)	BE(*H)-BE(*HOCO)	BE(*H)-BE(*CO)
**(PdAg)H**	1.428955	-0.884492	-0.273546	2.313447	1.702501
**(PdCu)H**	1.1945	-1.072389	-0.380284	2.266889	1.574784
**(PdNi)H**	0.475501	-1.548044	-0.771407	2.023545	1.246908
**PdH**	0.588421	-1.368428	-0.274902	1.956849	0.863323
**(PdCo)H**	-0.030392	-1.74406	-1.263788	1.713668	1.233396
**(PdPt)H**	0.125165	-1.706647	-0.717953	1.831812	0.843118

Supplementary Table 13. DFT calculated binding energies (in eV) of *H, *CO and *HOCO on various binding sites.

Intermediates	*H			*CO			*HOCO		BE(*H)-BE(*HOCO) (M sites)[a]
Configurations	top-Pd	top-M	hollow	top-Pd	top-M	hollow	top-Pd	top-M	
(PdAg)H	0.27	1.43	0.19	-0.27	-0.10	-0.19	-1.36	-0.88	2.31
(PdCu)H	0.50	1.19	0.38	-0.35	-0.38	-0.03	-1.43	-1.07	2.26
(PdNi)H	0.61	0.48	0.25	-0.33	-0.77	-0.03	-1.41	-1.55	2.03
PdH	0.59	-	0.42	-0.27	-	-0.18	-1.37	-	1.96
(PdCo)H	0.58	-0.03	0.17	-0.36	-1.26	-0.44	-1.40	-1.74	1.71
(PdPt)H	0.54	0.13	0.16	-0.55	-0.44	-0.71	-1.40	-1.71	1.84

[a] The BE(*H) at the top site was used to calculate BE(*H)-BE(*HOCO).

Supplementary Table 14. The ECSA values determined from CO-stripping (ECSA-CO) and $Pd(OH)_2$ reduction (ECSA-$Pd(OH)_2$) capacitances for the samples.

Entry	ECSA-CO (cm^2)	ECSA-$Pd(OH)_2$ (cm^2)
**PdAg**	10.48949	2.283727
**PdCu**	10.89659	3.16822
**PdNi**	8.357829	2.72392
**Pd**	7.483946	4.47054
**PdCo**	7.89056	3.68605
**PdPt**	8.38568	2.79072

Note that all of the ECSA values in the main text are on the basis of ones from the CO stripping method because this is more widely adopted for Pd- and Pt-based catalysts.

Supplementary Table 15. DFT calculated OH formation energies.

Entry	Formation Energy (eV)
**(PdAg)H**	-1.94
**(PdCu)H**	-2.24
**(PdNi)H**	-2.02
**PdH**	-1.16
**(PdCo)H**	-1.97
**(PdPt)H**	-1.64

Supplementary Table 16. DFT calculated difference in the Gibbs free energy change (Δ(ΔG), in eV) between *HOCO and *HCOO intermediates over PdH and (PdM)H.

Entry	Δ(ΔG)[a)]
**(PdAg)H**	0.20[b)]
**(PdCu)H**	0.08[b)]
**(PdNi)H**	0.00
**PdH**	-0.24

[a)] Negative Δ(ΔG) values indicate the formation of *HCOO is favorable over that of *HOCO.

[b)] Even though the *HCOO intermediate is slightly favored, the production of formic acid was negligible at high overpotentials, suggesting that both *HOCO and *HCOO pathways can promote CO production.

Supplementary Table 17. DFT calculated binding energies (in eV) of *H, *CO and *HOCO on PdH(100) and (PdNi)H(100) surfaces.

Intermediates	*H			*CO			*HOCO		BE(*H)-BE(*HOCO) (M sites)[a]
Configurations	bridge	top-Pd	top-M	bridge	top-Pd	top-M	top-Pd	top-M	
PdH(100)	0.01	0.29	-	-1.16	-1.14	-	-2.07	-	2.36
(PdNi)H(100)	-0.60	-0.08	-0.26	moved to Ni site	-1.55	-2.22	-2.81	-2.86	2.60

[a] The BE(*H) at the top site was used to calculate BE(*H)-BE(*HOCO).

Supplementary Notes

Supplementary Note 1. Catalytic performance comparison between Au/C and PdAg.

The Au/C had high values of FE(CO) of 70.3 and 73.2 % even at low potentials of -0.6 and -0.7 V_{RHE}, respectively (Supplementary Figure 4). However, Au/C lost its high catalytic selectivity of CO_2-to-CO rapidly with increasing cathodic potential: The FE(CO) for Au/C decreased to 61.9, 44.8 and 31.1 % at -0.9, -1.0 and -1.1 V_{RHE}, respectively. In comparison, the best bimetallic catalyst in the current study, PdAg, kept its high FE(CO) of 69.8, 63.3 and 59.5 % at -0.9, -1.0 and -1.1 V_{RHE}, respectively. Moreover, as displayed in Supplementary Figure 7, PdAg showed comparable or higher J(CO) at -1.0 and -1.1 V_{RHE} compared with Au/C (2.24 *vs.* 2.25 and 3.25 *vs.* 2.40 mA cm^{-2}, respectively).

Supplementary Methods

Electrochemical Measurements

A typical air-tight two-compartment cell was filled with CO_2-saturated 0.5M $NaHCO_3$ solution. In each compartment, 50 mL of the electrolyte was filled, leaving ~17 mL of empty space. Nafion 117, a proton exchange membrane, was used to separate catholyte and anolyte. The working electrode was placed together with reference electrode in the air-tight cathodic compartment while the counter electrode was located in the anodic compartment.

The electrochemical measurements were carried out using a Princeton Applied Research potentiostat. The applied potential was controlled by *i*R-compensation (80%) and converted to the reversible hydrogen electrode (V_{RHE}) by using the following Supplementary Equation (1):

$$V_{RHE} = V_{Ag/AgCl} + 0.205 + 0.059 \times pH \quad (1)$$

In order to attain stable electrochemical CO_2 reduction data, 10 cyclic voltammetry (CV) scans were first performed with a scan rate of 100 mV s^{-1} from 0.05 to 1.23 V_{RHE}. After that, the electrode was scanned from 0 to -1.2 V_{RHE} by using the linear scanning voltammetry (LSV) technique for three scans. Then, five additional CV scans were used to get rid of the products from the previous LSV step under CO_2 purging. After the additional CO_2 bubbling for 10 min, the electrochemical CO_2RR performance was evaluated by using the chronoamperometry (CA) method at each constant potential for a designated duration. The vigorous magnetic stirring was applied during the electrolysis to help mitigate the mass transport limitation of dissolved CO_2 in the electrolyte. With an increase in the applied potentials, the duration for CO_2RR was shortened in a such way that the amount of gas products (*i.e.*, CO and H_2) above the electrolyte would not exceed the calibration limitation of gas chromatography (GC,

Agilent, Agilent 7890B). Once each CA was completed for CO_2 RR, 100 μL of gas in the empty head space at the cathodic compartment was collected and injected into GC to quantify the gaseous products. The liquid products were quantified by using ^{1}H NMR spectra with an Avance III spectrometer (Bruker) operating at 400 MHz. Typically, 500 μL of electrolyte taken at the conclusion of the electrolysis was mixed with 10 μL of D_2O and 10 μL of internal standard solution. 2,2,3,3-d(4)-3-(Trimethylsilyl)propionic acid sodium salt (Alfa Aesar) was used as the internal standard (10 mM in D_2O). The ^{1}H NMR spectrum was measured in the water suppression mode. The peak corresponding to HCOOH was detected around a chemical shift of ~8.4 ppm. Before switching to another potential, the cathodic compartment in the cell was opened with CO_2 bubbling to purge dissolved CO gas in the electrolyte. Then, the cell was sealed again, followed by the next potential

***In-situ* XAFS Measurements**

The obtained spectra were processed using the ATHENA and ARTEMIS software in IFFEFIT package. The procedure[1] which was described in S. Kelly *et al.* was followed during the data process. For the reference, copper oxides ($Cu_2^{I}O$ and $Cu^{II}O$) were also measured together.

The areal mass loading of working electrode was *c.a.* 8.5 mg cm^{-2} in order to achieve sufficient XAFS signal. A laboratory-made H-shaped acryl cell was used for electrochemical operations. During the measurements, CO_2 gas was continuously bubbled into the electrolyte. The other conditions were the same as in the electrochemical measurements. The potential was scanned at a scan rate of 0.05 mV s^{-1} under LSV mode in a range from 0.1 to -0.6 V_{RHE}. Such a slow scan rate can minimize noise on the resultant spectra during *in-situ* XAFS analyses.

Extended X-ray Absorption Fine Structure (EXAFS) analyses were conducted by using the ARTEMIS software. The original EXAFS spectrum ($\chi(k)$) was weighted with k^2 value. The Hanning window was utilized for the Fourier-transform. All of the EXAFS fitting were done in the *R* space. The goodness of fitting was evaluated based on the reliable factor (R-factor) and reduced chi square (reduced χ^2). The fitting was done for the initial and final spectra from the XAFS analyses (Supplementary Figure 13). The fitting parameters are tabulated in Supplementary Table 2-10.

The typical Pd crystal structure (Space group=Fm-3m, lattice constant=3.89 Å) was utilized as the fitting model for the metallic phase. For the hydride phase, the rocksalt type PdH structure (Space group=Fm-3m, lattice constant=4.00 Å) was used. Single scattering path between Pd and M (M indicates the nearest neighbor atom around the Pd, M=Pd, Cu, Ag, Pt) was taken into consideration for all EXAFS fitting.

The Statistical Analysis on the Linearity Correlation.

The statistical analysis was performed for the linear correlation of $J_{ECSA}(CO/H_2)$ with the calculated ΔG. In the scale consideration between J_{ECSA} (from experimental results) and ΔG (from DFT calculation), we first excluded PdPt because it did not lead to hydride formation. Second, in the cases of correlating ΔG of each reaction intermediate and the corresponding J_{ECSA} values, the linear scaling between ΔG(*H) and $J_{ECSA}(H_2)$ was found (Supplementary Figure 20). Finally, in order to find out a descriptor that is able to describe both CO_2RR and HER, parameters combining both CO_2RR and HER (*i.e.*, ΔG(*H)-ΔG(*HOCO) or ΔG(*H)-ΔG(*CO)) were introduced. In this regard, even though the correlation between $J_{ECSA}(CO)$ and ΔG(*H)-ΔG(*CO) appear to be plausible (Supplementary Figure 22 and Supplementary Figure 23), ΔG(*H)-ΔG(*CO) cannot be a potential descriptor for CO_2RR because it cannot give

rise to a high linearity with $J_{ECSA}(H_2)$. Therefore, we reach a conclusion that the value of ΔG(*H)-ΔG(*HOCO) is the most reasonable descriptor for explaining both CO_2RR and HER.

Supplementary References

[1] Kelly, S., Hesterberg, D. & Ravel, B. Analysis of Soils and Minerals using X-ray Absorption Spectroscopy. *Methods Soil Anal.* **5**, 387-463 (2008).
